# Negotiating excess treatment costs in a clinical research trial: the good, the bad and the innovative

**DOI:** 10.1186/s13063-016-1208-5

**Published:** 2016-02-09

**Authors:** Rebecca Palmer, Madeleine Harrison, Elizabeth Cross, Pam Enderby

**Affiliations:** School of Health and Related Research, University of Sheffield, 107 Innovation Centre, 217, Portobello, Sheffield S1 4DP UK

**Keywords:** Excess treatment costs, Clinical trials, Trial set up

## Abstract

**Abstract:**

Barriers to recovering the excess treatment costs associated with health research from local organisations in the United Kingdom can increase research costs, delay completion of high- quality studies and risk disenfranchising health trusts and patients from participation. The authors demonstrate how the process for recovering excess treatment costs at a local National Health Service (NHS) trust level in a multicentre study was inconsistent and resulted in excess effort and cost to the research budget. An innovative example of how an organisation acting as a broker between commissioners and researchers facilitated a more timely excess treatment cost agreement is highlighted.

**Trial registration:**

Current Controlled Trials ISRCTN68798818, registered on 18 February 2014.

## Findings

### Attributing the costs of health and social care research and development (AcoRD)

The AcoRD guidance, introduced in May 2012, attributes the cost of research in the National Health Service (NHS) to research costs, NHS treatment costs or NHS support costs based on the primary purpose of the activity [1]. This guidance is specific to research being undertaken in the UK. As the NHS bears the cost of the patient care, the costs of the interventions within a research study are the NHS treatment costs. Where the cost of an experimental treatment is greater than the cost of usual care, it represents an ‘excess treatment cost’ (ETC), which is still viewed as part of the NHS treatment costs to be recovered through normal commissioning processes [2].

### Negotiating ETCs in the Big CACTUS study

The Big CACTUS study is a randomised controlled trial investigating the cost effectiveness of self-managed computer aphasia therapy compared with attention control or usual care for community-dwelling patients 4 months or more after the onset of a stroke. The intervention comprises computer exercises tailored to individual needs by a speech and language therapist (SLT) and support from an SLT/rehabilitation assistant or volunteer [3].

A previous pilot study indicated that 285 participants were required, necessitating 20 SLT departments to take part, 13 of which are in England [4]. Based on the AcoRD guidelines [1], as the trial intervention was being delivered in addition to usual care, the SLT time, assistant time, software and computers are all ETCs.

### The impact of local bureaucracy

**Fig. 1:**
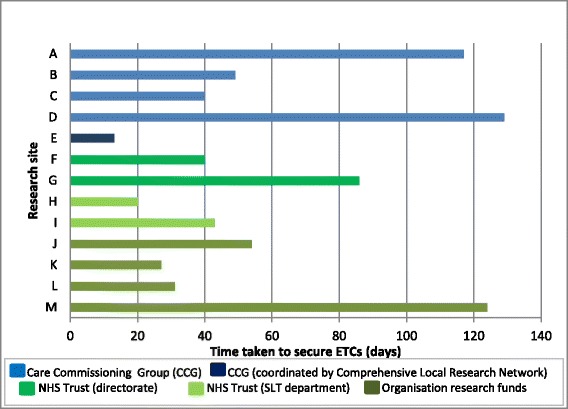
Who pays the excess treatment costs (ETCs)? How long does it take to secure a decision?

Local NHS organisations are responsible for recovering the NHS treatment costs associated with research. As independent legal entities, they can decide if and how to address this, which resulted in a myriad of different approaches to securing ETCs in the set-up of the Big CACTUS project (Fig. 1).

In eight NHS trusts, the excess treatment sum of £3,000 to 4,000 was considered too small to make an application to a Care Commissioning Group (CCG) (Fig. 1), and costs were met through trust reserves or directorates housing SLT departments (F and G) or through the SLT departments themselves (H and I). The ETCs were covered by research funds within local organisations delivering the intervention at sites J, K, L and M. This is notable, as AcORD suggests ETCs should be recovered through normal commissioning processes, which would not use research funds to support clinical activity.

The ETCs were met by CCGs in five sites (A, B, C, D, and E). In sites B and C, applications needed to be made to more than one CCG for them to cover the costs jointly. In site C, four CCGs were asked to share the cost (£750 each). This request was refused by one of the four CCGs, as they only wished to fund research into medicinal products. Commissioners rejected an application for ETCs at site H on the grounds that they ‘already pay enough for stroke care’. Where ETCs are above £20,000, per trust, application can be made for national subvention funding. However, because Big CACTUS required smaller sums, if the SLT departments were unable to absorb the ETCs, the trust and its patients would have been disenfranchised from participating in the study.

Figure 1 shows that the time taken for an organisation to make a decision to fund ETCs ranged from 13 days (2 weeks) to 124 days (18 weeks), with a mean of 59 days (8.5 weeks). This caused delay in getting research governance approvals and risked delaying the start of recruitment, with potential implications for recruiting to time and target.

The time taken to secure the ETCs for the study (approximately £70,000) required approximately 127 days of an experienced Clinical Trials Unit trial manager and research assistant time, representing a cost of £45,950 to the research budget. This time included identifying an individual who knew about ETCs at each Trust, understanding the local processes at each Trust, completing individual Trust application forms, making follow-up telephone calls, sending additional information and facilitating negotiations between R&D departments, Trust finance, directorates and SLT departments. Whilst we were unable to establish the exact time and cost of local SLT and R&D staff in the process, we estimate that these would be similar to those borne by the clinical trials unit. The total cost of the research time taken to negotiate excess treatment costs was therefore greater than the ETCs themselves for this study.

### Excess treatment cost brokers

The Hampshire & Isle of Wight Comprehensive Local Research Network (CLRN) and Thames Valley CLRN have adopted an approach whereby they manage an annual budget from the local commissioners for ETCs, thus being brokers between researchers and commissioners [5]. They proposed this model to provide standardised and timely funding agreements, thereby enabling studies with ETCs to be opened to recruitment without delay. The Big CACTUS site E was covered by these networks. One application was made by the research team to the CRN, and the ETCs were approved in 2 weeks, the shortest time of all the sites (Fig. 1).

## Conclusion

At a time where cost efficiency and improving patient care through high-quality research are key priorities, a lack of streamlined processes to recover ETCs can increase the cost of research, restrict Trust and patient participation and risk the delivery of high-quality studies. Identifying organisations who can be brokers between commissioners and researchers could reduce these barriers.

In November 2015, NHS England published further guidance on excess treatment costs to clarify the responsibilities of service commissioners and providers: https://www.england.nhs.uk/wp-content/uploads/2015/11/etc-guidance.pdf.

## Abbreviations

AcORD: Attributing the costs of health and social care research and development
CCG: Care Commissioning Group
CLRN: Comprehensive Local Research Network
ETC: Excess Treatment Costs
NHS: National Health Service
SLT: Speech and Language Therapist
